# High resolution CMR perfusion imaging demonstrates reduced flow reserve and endo/epi ratio in microvascular coronary disease

**DOI:** 10.1186/1532-429X-17-S1-P148

**Published:** 2015-02-03

**Authors:** Peter Shaw, Yang Yang, Yan Li, Jorge A Gonzalez, Angela M Taylor, Craig H Meyer, Frederick H Epstein, Michael Salerno, Christopher M Kramer

**Affiliations:** Biomedical Engineering, University of Virginia, Charlottesville, VA USA; Radiology, University of Virginia, Charlottesville, VA USA; Department of Medicine, Cardiology Division, University of Virginia, Charlottesville, VA USA

## Background

Patients with angina without obstructive coronary artery disease are increasingly recognized and microvascular disease (MVD) is felt to play a significant role. Patients with MVD have an abnormal myocardial perfusion reserve (MPR). An abnormal MPR by positron emission tomography is prognostic of increased cardiovascular events, particularly in women, diabetics, and those with metabolic syndrome. Also, high resolution CMR perfusion imaging has been shown to detect transmural differences in perfusion as evidenced by an endocardial-to-epicardial ratio (EER) >1 in normal segments and a reduced ratio in segments with obstructive CAD. We hypothesize that there would be a global reduction in MPR and EER with stress due to subendocardial ischemia in patients with anginal symptoms and non-obstructive CAD/normal coronaries.

## Methods

Patients with high suspicion for microvascular disease with angina or anginal equivalent symptoms with no significant epicardial stenosis as demonstrated by x-ray angiography underwent vasodilator stress perfusion studies on a 1.5T Siemens Avanto. High resolution first-pass spiral perfusion pulse sequences were performed. Sequence parameters included: 8 interleaves of variable density spirals from 0.75 to 0.2 Nyquist, 6.1ms readout per interleaf, TE 1.0 ms, TR 9ms, TI 80ms, FA 35, FOV 340mm, in-plane resolution 1.48mm. Images were acquired at 3 short axis slice locations during rest and stress (adenosine n=5, regadenoson n=8). Quantification of perfusion was performed on a pixel-wise basis using Fermi-function deconvolution after images were reconstructed by SPIRIT and then aligned with non-rigid registration ANTs (Advanced Normalization Tools).

## Results

13 patients (10 female, 3 male) completed the stress CMR protocol. Values are presented as mean ± SD. Rest flow was 1.27 ± 0.28 and stress flow was 2.44 ± 0.55 with a resultant MPR of 1.95 ± 0.42. Stress EER was lower than rest (0.95 ± 0.05 vs 1.02 ± 0.06, p = 0.002). There was no difference in flow between the epicardium and endocardium at rest; however there was a reduced EER (0.95 ± 0.05, p = 0.02) at stress. The MPR ratio from the endocardium to the epicardium was different between rest and stress and was less than 1 (0.94 ± 0.05, p= 0.001).

## Conclusions

Patients with microvascular disease and no significant epicardial coronary disease demonstrate reduced global MPR and EER ratios, suggesting that subendocardial ischemia plays a significant role in the disease process.

## Funding

UVA Center of Excellence Clinical Research Grant, T32 EB003841, K23 HL112910.

**Figure 1:**
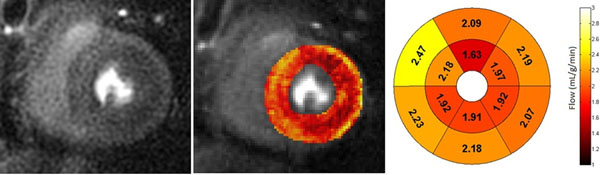
**Left: High resolution CMR perfusion image of a mid ventricular slice.** Center: Pixel based flow map during stress. Right: Corresponding regional subepicardial and subendocardial flow values.

**Figure:**
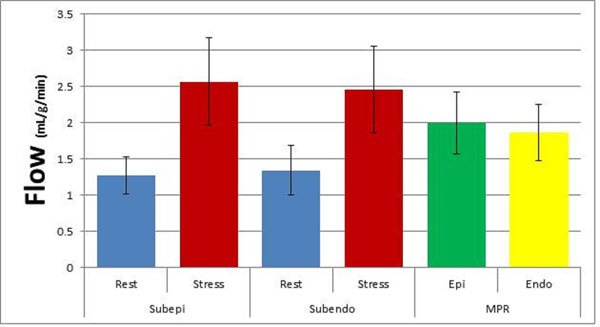
Figure 2

